# Boosted EfficientNet: Detection of Lymph Node Metastases in Breast Cancer Using Convolutional Neural Networks

**DOI:** 10.3390/cancers13040661

**Published:** 2021-02-07

**Authors:** Jun Wang, Qianying Liu, Haotian Xie, Zhaogang Yang, Hefeng Zhou

**Affiliations:** 1 Department of Informatics, King’s College London, London WC2R 2LS, UK; jun.1.wang@kcl.ac.uk; 2 College of Management, Shenzhen University, Shenzhen 518060, China; qianying_liu@163.com; 3 Department of Mathematics, The Ohio State University, Columbus, OH 43210, USA; xie.908@osu.edu; 4 Department of Radiation Oncology, University of Texas Southwestern Medical Center, Dallas, TX 75390, USA

**Keywords:** diagnosis, metastatic breast cancer, CNN, image classification, feature fusion

## Abstract

**Simple Summary:**

The assistance of computer image analysis that automatically identifies tissue or cell types has greatly improved histopathologic interpretation and diagnosis accuracy. In this paper, the Convolutional Neural Network (CNN) has been adapted to predict and classify lymph node metastasis in breast cancer. We observe that image resolutions of lymph node metastasis datasets in breast cancer usually are quite smaller than the designed model input resolution, which defects the performance of the proposed model. To mitigate this problem, we propose a boosted CNN architecture and a novel data augmentation method called Random Center Cropping (RCC). Different from traditional image cropping methods only suitable for resolution images in large scale, RCC not only enlarges the scale of datasets but also preserves the resolution and the center area of images. In addition, the downsampling scale of the network is diminished to be more suitable for small resolution images. Furthermore, we introduce attention and feature fusion mechanisms to enhance the semantic information of image features extracted by CNN. Experiments illustrate that our methods significantly boost performance of fundamental CNN architectures, where the best-performed method achieves an accuracy of 97.96% ± 0.03% and an Area Under the Curve (AUC) of 99.68% ± 0.01% in Rectified Patch Camelyon (RPCam) datasets, respectively.

**Abstract:**

(1) Purpose: To improve the capability of EfficientNet, including developing a cropping method called Random Center Cropping (RCC) to retain the original image resolution and significant features on the images’ center area, reducing the downsampling scale of EfficientNet to facilitate the small resolution images of RPCam datasets, and integrating attention and Feature Fusion (FF) mechanisms with EfficientNet to obtain features containing rich semantic information. (2) Methods: We adopt the Convolutional Neural Network (CNN) to detect and classify lymph node metastasis in breast cancer. (3) Results: Experiments illustrate that our methods significantly boost performance of basic CNN architectures, where the best-performed method achieves an accuracy of 97.96% ± 0.03% and an Area Under the Curve (AUC) of 99.68% ± 0.01% on RPCam datasets, respectively. (4) Conclusions: (1) To our limited knowledge, we are the only study to explore the power of EfficientNet on Metastatic Breast Cancer (MBC) classification, and elaborate experiments are conducted to compare the performance of EfficientNet with other state-of-the-art CNN models. It might provide inspiration for researchers who are interested in image-based diagnosis using Deep Learning (DL). (2) We design a novel data augmentation method named RCC to promote the data enrichment of small resolution datasets. (3) All of our four technological improvements boost the performance of the original EfficientNet.

## 1. Introduction

Even though considerable advances have been made in understanding cancers and implementing the diagnostic and therapeutic methods, breast cancer is the most common malignant cancer diagnosed globally and is the secondary leading cause of cancer-associated death in women [1,2,3]. Metastatic Breast Cancers (MBCs), the main cause of death from incurable breast cancer, spreads from local invasion of peripheral tissue to lymphatic and blood vessels and ends in distant organs [4,5,6]. It is estimated that 10 to 50% of patients experience metastases eventually, despite being diagnosed with regular breast cancer at the beginning [7]. Moreover, because of the primary tumor subtype, the metastasis rate and site are heterogeneities [8]. Thus, prognosis, accurate diagnosis, and treatment for MBCs remain challenging. For MBC diagnosis, one of the most important tasks is the staging of BC that counts the recognition of Axillary Lymph Node (ALN) metastases, which is detectable among most node-positive sufferers using Sentinel Lymph Node (SLN) biopsies [9,10]. Assessing microscopy images from SLNs are conventional techniques for evaluating ALNs. However, they require on-site pathologists to investigate samples, which is time-consuming, laborious, and less reliable due to a certain degree of subjectivity, particularly in cases that contain small lesions or in which the lymph nodes are negative for cancer [11].

Consequently, developing digital pathology methods to assist in microscopic diagnosis has evolved significantly during the last decade [12,13]. Advanced scanning technology, cost reduction, quality of spatial images, and magnification have made full digitalization feasible for evaluating histopathologic tissues [14]. Multiple advantages appear with digital pathology technology development, which include online consultation and case analysis, thus advancing the availability of samples and waiving on-site experts. However, manual inspection is also necessary, and the potential inconsistent diagnosis decisions may affect the accuracy of diagnosis. In addition, hospitals are often short of advanced equipment and pathologists to support digital pathology. It is reported that presumptive treatment phenomena may exist widely among developing countries due to the lack of well-trained pathologists and advanced equipment [15]. Moreover, the majority of the population often has difficulty getting access to pathology and laboratory medicine services. Regarding cancer, cardiovascular disease, and bone generation as examples, few communities can get the pathology and laboratory medicine treatment [16,17,18,19,20,21].

To better facilitate digital pathology and alleviate the above mentioned problems, various analytic approaches have been proposed (e.g., deep learning, machine learning, and some specific software) to strengthen the accuracy and sensitivity of metastatic cancer detection [22,23,24,25,26]. With excellent robust ability to extract features in images, a Convolutional Neural Network (CNN) becomes the most successful deep learning-based method in the Computer Vision (CV) field. It has been widely used in diseases diagnosed with microscopy (e.g., Alzheimer’s disease) [27,28,29,30]. CNN automatically learns image features from multiple dimensions in a large image dataset, which is applied to identify or classify structures and is therefore applicable in multiple automated image-recognition biomedical areas [31,32]. CNN-based cancer detection has proved to be a convenient method to classify tumors from other cells or tissues and has demonstrated satisfactory results [33,34,35,36]. EfficientNet is one of the most potent CNN architectures. It utilizes a compound scaling method to enlarge the network depth, width, and resolution, obtaining state-of-the-art capacity in various benchmark datasets while requiring fewer computation resources than other models [37]. Hence, the EfficientNet is a suitable model, which may provide significant medical image classification potentials, although there is a substantial difference between medical images and traditional images. However, less attention has been paid to the abilities of EfficientNet in medical images, making motivation for us to conduct this work.

One core problem defecting the performance of these CNN-based models in medical imaging is the image resolution disparity. State-of-the-art CNN models normally are designed for large resolution images (e.g., 500 × 500 or larger), but the image resolution of the lymph node metastasis datasets in breast cancer is usually quite smaller (e.g., 96 × 96 If we follow the same CNN structure designed for large resolution images to process the small resolution medical images, the final extracted features could be too abstractive to classify. In addition, before sending training images to models, the data augmentation method cropping is utilized to uniform input resolution (e.g., 224 × 224) and enrich the dataset. The performance of Deep Learning (DL) models relies heavily on the scale and quality of training datasets since a large dataset allows researchers to train deeper networks and improves the generalization ability of models, thus enhancing the performance of DL methods. However, traditional cropping methods, such as center cropping and random cropping, cannot be simply applied since they will further reduce the image size. Moreover, the discriminative features to detect the existence of cancer cells usually concentrate on the center areas of images on some datasets, and traditional cropping methods may lead to the loss and incompleteness of these informative areas.

To cope with the aforementioned problems, we propose three strategies to improve the capability of EfficientNet, including developing a cropping method called Random Center Cropping (RCC) to retain the original image resolution and discriminative features on the center area of images, reducing the downsampling scale of EfficientNet to facilitate the small resolution images of Rectified Patch Camelyon (RPCam) datasets, and integrating the attention and FF mechanisms with EfficientNet to obtain features containing rich semantic information. This work has three main contributions: (1) To our limited knowledge, we are the first study to explore the power of EfficientNet on MBC classification, and elaborate experiments are conducted to compare the performance of EfficientNet with other state-of-the-art CNN models, which might inspire those who are interested in image-based diagnosis using Deep Learning (DL); (2) A new data augmentation method, RCC, is investigated to promote the data enrichment of datasets with small resolution; (3) These four technical improvements noticeably advance the performance of the original EfficientNet. The best accuracy and Area Under the Curve (AUC) achieve 97.96% ± 0.03% and 99.68% ± 0.01%, respectively, confirming the applicability of utilizing CNN-based methods for MBC diagnosis.

## 2. Results

### 2.1. Summary of Methods

Rectified Patch Camelyon (RPCam) was used as the benchmark dataset in our study to verify the performance of our proposed methods for detecting BC’s lymph node metastases. We utilized the original EfficientNet-B3 as the baseline binary classifier to implement our ideas. Firstly, the training and testing performances of boosted EfficientNet were evaluated and compared with two state-of-the-art backbone networks called ResNet50 and DenseNet121 [38] and the baseline model. To investigate the capability of each strategy (Random Center Cropping, Reduce the Downsampling Scale, Feature Fusion, and Attention) adopted in the boosted EfficientNet, ablation studies were conducted to explore the performance of the baseline network combining with a single strategy and multiple strategies.

### 2.2. The Performance of Boosted EfficientNet-B3

**Figure 1:**
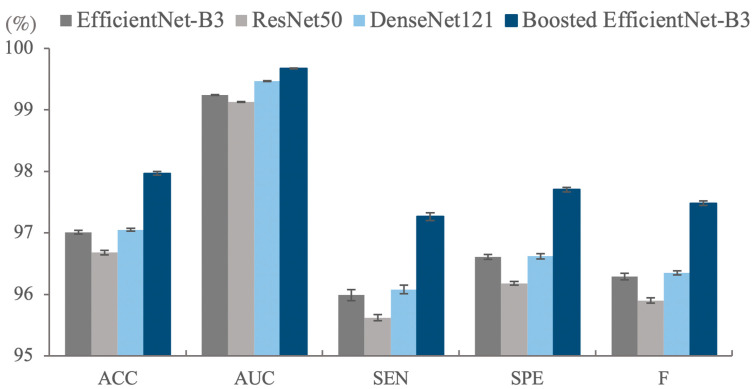
The classification results (%) of different methods on the test set of the Rectified Patch Camelyon (RPCam) dataset. Boosted EfficientNet-B3 outperforms the other three models in all evaluation metrics. Error bars represent standard deviation errors. ACC, Accuracy; AUC, Area Under the Curve; SEN, Sensitivity; SPE, Specificity; F, F1-Measure.

As illustrated in Table 1 and Figure 1, the basic EfficientNet outperforms the boosted-EfficientNet-B3 on the training set both on the Accuracy (ACC) and AUC, while a different pattern can be seen when applying them on the testing set. The contradictory trend is because the basic EfficientNet overfits the training set, while the boosted-EfficientNet-B3 mitigates overfitting problems since RCC enables the algorithm to crop images randomly, thus improving the diversity of training images. Although enhancing the performance of a well-performing model is of great difficulty, the boosted-EfficientNet-B3 significantly improves the ACC from 97.01% ± 0.03% to 97.96% ± 0.03% and noticeably boosts AUC from 99.24% ± 0.01% to 99.68% ± 0.01% compared with the basic EfficientNet-B3. Furthermore, more than a 1% increase can be seen in the Sensitivity (SEN), Specificity (SPE), and F1-Measure (F).

Similar patterns of comparison can be found when comparing EfficientNet-B3 to other CNN architectures. Notably, ResNet50 and DenseNet121 suffer from the overfitting problem severely. EfficientNet-B3 obtains better performance than ResNet50 and DenseNet121 for all indicators on the testing dataset while using fewer parameters and computation resources, as shown in Figure 1. All these results confirm the capability of our methods, and we believe these methods can boost other state-of-the-art backbone networks. Therefore, we intend to extend the application scope of these methods in the future. Ablation studies were conducted to illustrate the effectiveness and coupling degree of the four methods, which are elaborated in Section 2.3.

### 2.3. Ablation Studies

To specifically handle the MBC task of which the data resolution is small, we adopted four strategies, including Random Center Cropping (RCC), Reduce the Downsampling Scale (RDS), FF, and Attention, on the baseline model, which is also the difference between our work and predecessors. In this part, we conducted ablation experiments to illustrate the capacity of each strategy. We utilized AUC and ACC as primary metrics to evaluate the performance of the model.

The results reveal that these four key strategies contribute to the cancer detection, including increased generalizability and higher accuracy to the classifier models. Specifically, the inclusion of RCC augments the datasets and retains the most informative areas, leading to increased generalizability to unseen data. In addition, RDS improves feature representation ability by adjusting the excessive downsampling multiple to a suitable scale. Simultaneously, FF and Attention mechanisms effectively improve the feature representation ability and increase the response of vital features.

#### 2.3.1. The Influence of Random Center Cropping

From the first two rows of Table 2, it can be observed that the RCC significantly boosts the performance of the algorithms by noticing the AUC increases from 99.24 to 99.54%, and the ACC increases from 97.01 to 97.57% because RCC enhances the diversity of training images and mitigates the overfitting problem.

#### 2.3.2. The Influence of Reducing the Downsampling Scale

As the first and third rows of Table 2 show, modest improvements in ACC and AUC (0.35 and 0.19%, respectively) are achieved because of the larger feature map. The image resolution of the RPCam dataset is much lower than the designed input of the EfficientNet-B3, resulting in smaller and abstractive features, thus adversely affecting the performance. It is worth noting that the improvement of the RDS is enhanced when being combined with the RCC.

#### 2.3.3. The Influence of Feature Fusion

FF combines low-level and high-level features to boost the performance of models. As the results in Table 2 indicate, when adopting only one mechanism, the FF demonstrates the largest AUC and the second-highest ACC increases among RCC, RDS, and FF, revealing FF’s adaptability and effectiveness in EfficientNet. The FF contributes to more remarkable improvement to the model after utilizing RCC and RDS since ACC reaches the highest value, and AUC comes to the second-highest among all methods.

#### 2.3.4. The Influence of the Attention Mechanism

Combining the attention mechanism with FF is critical in our work. Utilizing the attention mechanism to enhance the response of cancerous tissues and suppress the background can further boost the performance. From the fourth and fifth rows of Table 2, it can be seen that the attention mechanism improves the performance of original architectures both in the ACC and AUC, confirming its effectiveness. Then, we analyzed the last four rows. When the first three strategies were employed, adding attention increases the AUC by 0.02%, but the ACC remains at a 97.96% value. Meanwhile, attention brings a significant performance improvement compared with models that only utilize RCC and FF, since ACC and AUC are increased from 97.59 to 97.85% and from 99.58 to 99.68%, respectively. Although the model using all methods demonstrates the same value of the AUC as the model only utilizing RCC, RDS, and FF, all utilized models have a 0.11% ACC improvement. A possible reason for the minor improvement between these two models is that RDS enlarges the size of the final feature maps, thus maintaining some low-level information, which is similar to the FF and attention mechanism.

## 3. Discussion

With the rapid development of computer vision technology, computer hardware, and big data technology, image recognition based on DL has matured. Since AlexNet [39] won the 2012 ImageNet competition, an increasing number of decent ConvNets have been proposed (e.g., VGGNet [40], Inception [41], ResNet [42], DenseNet [43]), leading to significant advances in computer vision tasks. Deep Convolutional Neural Network (DCNN) models can automatically learn image features, classify images in various fields, and possess higher generalization ability than traditional Machine Learning (ML) methods, which can distinguish different types of cells, allowing the diagnosis of other lesions. This technology has also achieved remarkable advances in medical fields. In past decades, many articles have been published relevant to applying the CNN method to cancer detection and diagnosis [44,45,46,47].

CNNs have also been widely developed in MBC detection. Agarwal et al. [48] released a CNN method for automated masses detection in digital mammograms, which used transfer learning with three pre-trained models. In 2018, Ribli et al. proposed a Faster R-CNN model-based method for the detection and classification of BC masses [49]. Furthermore, Shayma’a et al. used AlexNet and GoogleNet to test BC masses on the National Cancer Institute (NCI) and Mammographic Image Analysis Society (MIAS) database [38]. Furthermore, Al-Antari et al. presented a DL method, including detection, segmentation, and classification of BC masses from digital X-ray mammograms [50]. They utilized the CNN architecture You Only Look Once(YOLO) and obtained a 95.64% accuracy and an AUC of 94.78% [51]. EfficientNet, a state-of-the-art DCNN, that maintains competitive performance while requiring remarkably lower computation resources in image recognition is proposed [37]. Great successes could be seen by applying EfficientNet in many benchmark datasets and medical imaging classifications [52,53]. This work also utilizes EfficientNet as the backbone network, which is similar to some aforementioned works, but we focus on the MBC task. There are eight types of EfficientNet, from EfficientNet-B0 to EfficientNet-B7, with an increasing network scale. EfficientNet-B3 is selected as our backbone network due to its superior performance over other architectures according to our experimental results on RPCam datasets. In addition, quite differently from past works that usually use BC masses datasets with large resolution, our work detects the lymph node metastases in breast cancer, and the dataset resolution is small. To our limited knowledge, we are the first researchers to utilize EfficientNet to detect lymph node metastases in BC. Therefore, this work aims to examine and improve the capacity of EfficientNet for BC detection.

This study has proposed four strategies, including RCC, Reducing Downsampling Scale, Attention, and FF, to improve the accuracy of the boosted EfficientNet on the RPCam datasets. Discriminative features used for metastasis distinguishing are mainly focused on the central area (32 × 32) in an image, so traditional cropping methods (random cropping and center cropping) cannot be simply applied to this dataset as they may lead to incompleteness or even loss of these essential areas. Therefore, a method named Random Center Cropping (RCC) is investigated to ensure the integrity of the central 32 × 32 area while selecting peripheral pixels randomly, allowing dataset enrichment. Apart from retaining the significant center areas, RCC maintains more pixels enabling deeper network architectures.

Although EfficientNet has demonstrated competitive functions in many tasks, we observe a large disparity in image resolution between the designed model inputs and RPCam datasets. Most models set their input resolution to 224 ×224 or larger, maintaining a balance between performance and time complexity. The depth of the network is also designed for adapting the input size. This setting performs well in most well-known baseline image datasets (e.g., ImageNet [54], PASCAL VOC [55]) as their resolutions usually are more than 1000 × 1000. However, the resolution of RPCam datasets is 96 ×96, which is much less than the designed model inputs of 300 × 300. After the feature extraction, the size of the final feature will be 32 times smaller than the input (from 96 × 96 to 3 × 3). This feature map is likely to be too abstractive and thus lose low-level features, which may adversely affect the performance of EfficientNet. Hence, along with the RCC, we proposed to reduce the downsampling scale to mitigate this problem, and the experimental results prove our theory.

**Figure 2:**
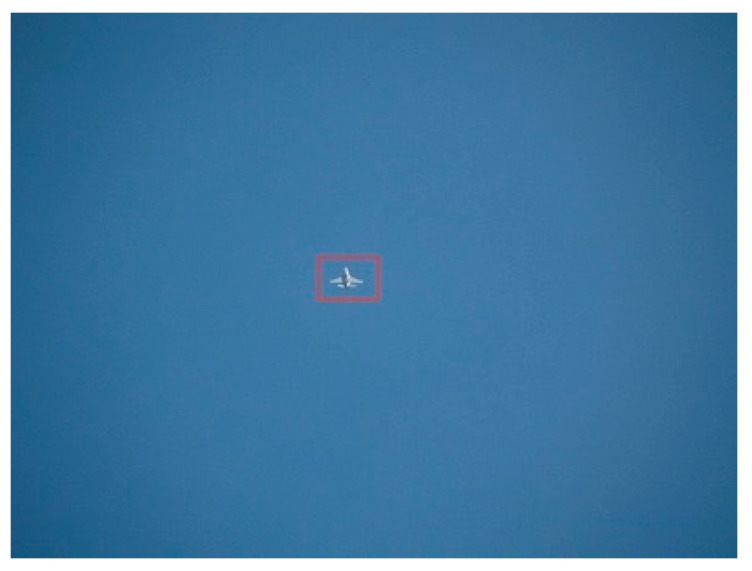
Attention in the human visual system. Although the image primarily involves the background, people are easily able to capture the airplane in the red rectangle.

When viewing a picture, the human visual system tends to selectively focus on a specific part of the picture while ignoring other visible information due to limited visual information processing resources. For example, although the sky information primarily covers Figure 2, people are readily able to capture the airplane in the image [55]. To simulate this process in artificial neural networks, the attention mechanism is proposed and has many successful applications including image caption [56,57], image classification [58], and object detection [59,60]. As previously stated, for RPCam datasets, the most informative features are concentrated in the center area of images, making attention to this area more critical. Hence, this project also adopts the attention mechanism implemented by a Squeeze-and-Excitation block proposed by Hu et al. [61].

Moreover, high-level features generated by deeper convolutional layers contain rich semantic information, but they usually lose details such as positions and colors that are helpful in the classification. In contrast, low-level features include more detailed information but introduce non-specific noise. FF is a technique that combines low-level and high-level features and has been adopted in many image recognition tasks for performance improvement [62]. Detailed information is more consequential in our work since complex texture contours exist in the RPCam images despite their small resolution. Accordingly, we adopt the FF technique to boost classification accuracy.

The experimental results reveal that boosted EfficientNet-B3 alleviates the problem of overfitting training images and outperforms the ResNet50, DenseNet121, and the basic EfficientNet-B3 for all indicators in testing datasets. Furthermore, the results of the ablation experiment indicate that these four strategies adopted are all helpful to enhance the performance of the classifier model, including generalization ability, accuracy, and computational cost.

There are some limitations in this work. Our main purpose was to propose a method to classify the lymph node metastases in BC, and we only tested the RPCam dataset. If multiple sources are applied for training, there are potentials to improve the model classification and generalization performance. Additionally, we believe our model can be used for other biomedical diagnostic applications after a few modifications.

Besides, we select features from the 4th, 7th, 17th, and 25th blocks to perform feature fusion, but other combinations may obtain better performance. Due to the limited computation resources, we have not tried other attention mechanisms and feature fusion strategies yet.

## 4. Materials and Methods

### 4.1. Rectified Patch Camelyon Datasets

**Figure 3:**
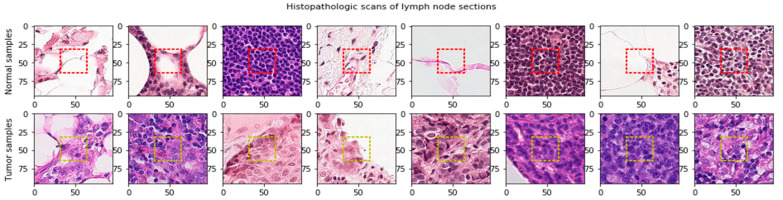
Lymph node sections extracted from digital histopathological microscopic scans. The significant features determine the cancer cells are in the center area (32 × 32). Red dash lines define the center region in the normal samples. Yellow dash lines define the center region in the tumor samples.

A Rectified Patch Camelyon (RPCam) dataset created by deleting duplicate images in the PCam dataset [63] was sponsored by the Kaggle Competition. The dataset consisted of digital histopathology images of lymph node sections from breast cancer. These images are in the size of 96 × 96 pixels and have 3 channels representing RGB (Red, Green, Blue) colors, and some of which are shown in Figure 3.

More importantly, these images are associated with a binary label for the positive (1) or negative (0) of breast cancer metastasis. In addition, the potential pathological features for classifying the cancerous tissues are in the center area with 32 × 32-pixel in size, as shown in the red dashed square of Figure 3. The RPCam data set consists of positive (1) and negative samples (0) with unbalanced proportions; 130,908 images in the positive class and 89,117 in the negative one.

### 4.2. Random Center Cropping

**Figure 4:**
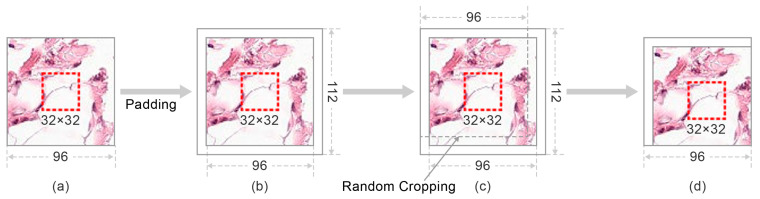
The workflow of the Random Center Cropping (RCC). (**a**) is the original training image. Images are first padded with eight pixels from four directions (**left**, **right**, **up**, **down**) to create a 112 × 112 resolution as shown in (**b**). (**c**) demonstrates the process that Random cropping is then performed on these modified images to restore a 96 × 96 resolution image (**d**). Particular center areas are shown in the red dashed rectangular and retained after the cropping process.

We denote I $\in {\mathbb{R}}^{96\times 96\times 3}$ as a training image in the RPCam dataset. As Figure 4 illustrates, RCC first enlarges image I by padding 8 pixels around the images. The padded images $I_{pad}=Padding\left(I,8\right),\;I_{pad}\in {\mathbb{R}}^{112\times 112\times 3}$ have random cropping performed to enrich the datasets. The resolution of the cropped image $I_{crop}=RandomCrop\left(I_{pad},\;96\times 96\right),\;\;I_{crop}\in {\mathbb{R}}^{96\times 96\times 3}$ returns to the original size. Eventually, these $I_{crop}$ images are fed as inputs into the CNN models to perform feature extraction and cancer detection. Despite enriching the dataset and improving the generalization ability of models, RCC guarantees the integrity of the center 32 × 32 areas in each $I_{crop}$ in the training set. As mentioned in Section 4.1, the potential pathological features for classifying the cancerous tissues are in the center area with 32 × 32 size. Hence, retaining the integrity of these areas may contribute positively to the models’ capability since training images contain informative patches rather than background noises.

### 4.3. Boosted EfficientNet

**Figure 5:**
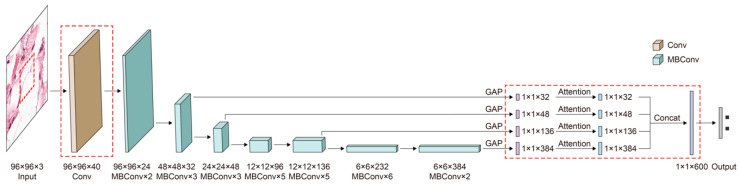
The architecture of boosted-EfficientNet-B3. EfficientNet first extracts image features through its convolutional layers. The attention mechanism is then utilized to reweight features, increasing the activation of significant parts. Next, we perform FF on the outputs of several convolutional layers. Subsequently, images are classified based on those fused features. Details of these methods are described in the following sections.

The architecture of boosted EfficientNet-B3 is shown in Figure 5. The main building block is MBConv [64,65]. The components in red dashed rectangles are different from the original EfficientNet-B3. Images are first sent to some blocks containing multiple convolutional layers to extract image features. Then, these features are weighted by the attention mechanism to improve the response of features contributing to classification. Next, the FF mechanism is utilized, enabling features to retain some low-level information. Consequently, images are classified according to those fused features.

### 4.4. Reduce the Downsampling Scale

To mitigate the problem mentioned in the discussion, we adjusted the downsampling multiple in EfficientNet. Our idea is implemented by modifying the stride of the convolution kernel of EfficientNet. To select the best-performed downsampling scale, multiple and elaborate experiments were conducted on the downsampling scale {2, 4, 6, 8, 16}, and Strategy 16 outperforms other settings. The size of the feature map in the best-performing downsampling scale (16) was 6 × 6, which is one times larger than the original downsampling multiple (32). The change of the downsampling scale from 32 to 16 was implemented by modifying the stride of the first convolution layer from two to one, as shown in the red dashed squares on the left half of Figure 5.

### 4.5. Attention Mechanism

**Figure 6:**
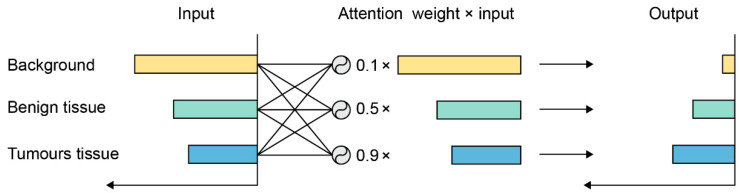
An example of the attention mechanism. The response to input is reweighted by the attention mechanism.

As an example for the attention mechanism, it can be seen from Figure 6 that the response to the background is large, since most parts of the image consist of the background. However, this information usually is useless for classification, so their response should be suppressed. On the other hand, cancerous tissue is more informative and deserves higher activation, so its response is enhanced after being processed by the attention mechanism.

We adopted the attention mechanism implemented by a Squeeze-and-Excitation block proposed by Hu et al. [61]. Briefly, the essential components are the Squeeze and Excitation. Suppose feature maps U have C channels and the size of the feature in each channel is H∗W. For the Squeeze operation, global average pooling is applied to U, enabling features to gain a global receptive field. After the Squeeze operation, the size of feature maps U change from H∗W∗C to 1∗1∗C. Results are denoted as Z. More precisely, this change is given by

(1) $$Z_{c}=F_{sq}\left(U_{c}\right)=\frac{1}{H\times W}\overset{W}{\underset{i=1}{\sum }}\overset{H}{\underset{j=1}{\sum }}U_{c}\left(i,j\right)$$

where c denotes $c^{th}$ channel of U, and $F_{sq}$ is the Squeeze function.

Following the Squeeze operation, the Excitation operation is to learn the weight (scalar) of different channels, which is simply implemented by the gating mechanism. Specifically, two fully connected layers are organized to learn the weight of features and activation function sigmoid, and Rectified Linear Unit (RELU) are applied for non-linearity increasing. Excepting the non-linearity, the sigmoid function also certifies the weight falls in the range of [0,1]. The calculation process of the scalar (weight) is shown in Equation (2).

(2) $$S=F_{ex}\left(Z,W\right)=\sigma \left(g\left(Z,W\right)\right)=\sigma \left(W_{2}\delta \left(W_{1}Z\right)\right)$$

where S is the result of the Excitation operation, $F_{ex}$ is the Excitation function, and g refers to the gating function. σ and δ denote the sigmoid and RELU function, respectively. $W_{1}$ and $W_{2}$ are learnable parameters of the two fully connected layers. The final output is calculated by multiplying the scalar S with the original feature maps U.

In our work, the attention mechanism is combined with the FF technique, as shown in Figure 5.

### 4.6. Feature Fusion

**Figure 7:**
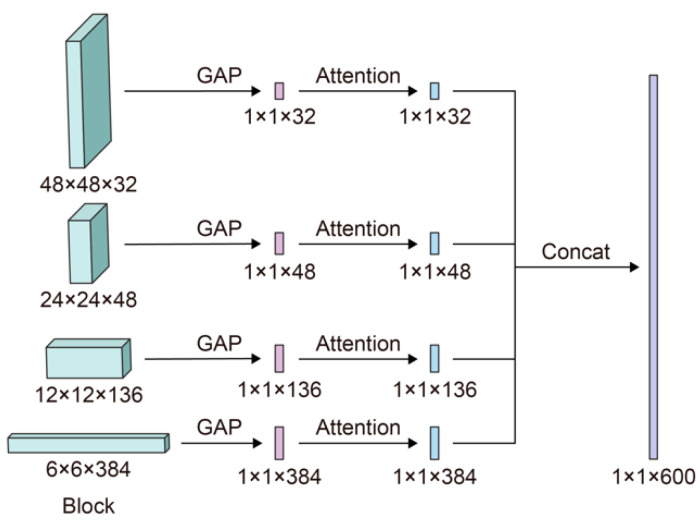
The approach to integrate the attention and FF mechanisms to EfficientNet-B3.

Four steps are involved during the FF technique, as shown in Figure 7. (1) During the forward process, we save the outputs (features) of the convolutional layers in the 4th, 7th, 17th, and 25th blocks. (2) After the last convolutional layer extracts features, the attention mechanism is applied to features recorded in Step 1 to value the essential information. (3) Low-level and high-level features are combined using the outputs of Step 2 after applying the attention mechanism. (4) These fused features are then sent to the following layers to conduct classification.

### 4.7. Evaluation Metrics

We evaluated our method on the RPCam dataset. Since the testing set was not provided, we split the original training set into a training set and a validation set and utilized the validation set to verify models. In detail, the capacities of models were evaluated by five indicators, including AUC, Accuracy (ACC), Sensitivity (SEN), Specificity (SPE), and F1-Measure [66]. AUC considers both Precision and Recall, thus comprehensively reflecting the performance of a model. The value of AUC falls into the range 0.5 and 1. A higher value indicates better performance. SEN represents the proportion of all positive examples that are correctly classified and measures the ability of classifiers to recognize positive examples, whereas SPE evaluates the ability of algorithms to recognize negative ones. Like the AUC, the F1-Measure considers Precision and Recall and is calculated by the weighted average of Precision and Recall. All indicators are calculated based on four fundamental indicators: True Positive (TP), True Negative (TN), False Positive (FP), False Negative (FN). The specific calculation processes are shown in Equations (3)–(6).

(3) $$ACC=\frac{TP+TN}{TP+FP+TN+FN}$$

(4) $$SEN\;=\frac{TP}{TP\;+\;FN}$$

(5) $$SPE\;=\frac{TN}{TN\;+\;FP}$$

(6) $$Fmeasure=\frac{2TP}{2TP+FN+FP}$$

### 4.8. Implementation Details

Our method is built on the EfficientNet-B3 model and implemented based on the PyTorch DL framework using Python [67]. Four pieces of GTX 2080Ti GPUs were employed to accelerate the training. All models were trained for 30 epochs. The gradient optimizer was Adam. Before being fed into the network, images were normalized according to the mean and standard deviation on their RGB-channels. In addition to the RCC, we also employed random horizontal and vertical flipping in the training time to enrich the datasets. During the training, the initial learning rate was 0.003, which was decayed by a factor of 10 at the 15th and 23rd epochs. The batch size was 256. The parameters of the boosted EfficientNet and other comparable models were placed as close as possible to enhance the credibility of the comparison experiment. In detail, the parameter sizes of these three models were increased in turn from the boosted EfficientNet, DenseNet121, and ResNet50.

## 5. Conclusions

The purpose of this project was to facilitate the development of digital diagnosis in MBCs and explore the applicability of a novel CNN architecture EfficientNet on MBC. In this paper, we proposed a boosted EfficientNet CNN architecture to automatically diagnose the presence of cancer cells in the pathological tissue of breast cancers. This boosted EfficientNet alleviates the small image resolution problem, which frequently occurs in medical imaging. Particularly, we developed a data augmentation method, RCC, to retain the most informative parts of images and maintain the original image resolution. Experimental results demonstrate that this method significantly enhances the performance of EfficentNet-B3. Furthermore, RDS was designed to reduce the downsampling scale of the basic EfficientNet by adjusting the architecture of EfficientNet-B3. It further facilitates the training on small resolution images. Moreover, two mechanisms were employed to enrich the semantic information of features. As shown in the ablation studies, both of these methods boost the basic EfficientNet-B3, and more remarkable improvements can be obtained by combining some of them. Boosted-EfficientNet-B3 was also compared with another two state-of-the-art CNN architectures, ResNet50 and DenseNet121, and shows superior performance. It can be expected that our methods can be utilized in other models and lead to improved performance of other disease diagnoses in the near future. In summary, our boosted EfficientNet-B3 obtains an accuracy of 97.96% ± 0.03% and an AUC value of 99.68% ± 0.01%, respectively. Hence, it may provide an efficient, reliable, and economical alternative for medical institutions in relevant areas.

## Figures and Tables

**Table 1:** Classification results (%) of different methods for the RPCam dataset.

	Training		Test				
	ACC	AUC	ACC	AUC	SEN	SPE	F
EfficientNet-B3	99.61 ± 0.02	99.99 ± 0.00	97.01 ± 0.03	99.24 ± 0.01	95.99 ± 0.09	96.61 ± 0.04	96.29 ± 0.05
ResNet50	99.85 ± 0.02	100.00 ± 0.00	96.68 ± 0.04	99.13 ± 0.01	95.62 ± 0.05	96.18 ± 0.03	95.90 ± 0.05
DenseNet121	99.78 ± 0.03	100.00 ± 0.00	97.05 ± 0.03	99.47 ± 0.01	96.08 ± 0.07	96.62 ± 0.04	96.35 ± 0.04
Boosted EfficientNet-B3	98.02 ± 0.03	99.74 ± 0.01	97.96 ± 0.03	99.68 ± 0.01	97.29 ± 0.06	97.65 ± 0.04	97.47 ± 0.04

**Table 2:** Classification performance comparison of EfficientNet with various strategies of the RPCam testing set.

	RCC	RDS	FF	Attention	ACC (%)	AUC (%)
EfficientNet					97.01	99.24
	√				97.57	99.54
		√			97.36	99.43
					97.55	99.57
				√	97.63	99.63
	√	√			97.73	99.62
	√	√	√		97.96	99.66
	√	√	√	√	97.96	99.68
	√		√		97.59	99.58
	√		√	√	97.85	99.68

## Data Availability

All source data relating to this manuscript are available upon request.
